# Atypical integration of social cues for orienting to gaze direction in adults with autism

**DOI:** 10.1186/2040-2392-6-5

**Published:** 2015-01-26

**Authors:** Chris Ashwin, Jari K Hietanen, Simon Baron-Cohen

**Affiliations:** Department of Psychiatry, Autism Research Centre, University of Cambridge, Douglas House, 18b Trumpington Rd, Cambridge, CB2 8AH UK; Department of Psychology, University of Bath, Claverton Down, Bath, BA2 7AY UK; Human Information Processing Laboratory, School of Social Sciences and Humanities/Psychology, University of Tampere, FI-33014 Tampere, Finland; Cambridgeshire and Peterborough NHS Foundation Trust, CLASS Clinic, Fulbourn Hospital, Cambridge, CB21 5EF UK

**Keywords:** Gaze, Attention, Social orienting, Theory of mind, Asperger syndrome, Autism, Autism spectrum conditions

## Abstract

**Background:**

Gaze direction provides important information about social attention, and people tend to reflexively orient in the direction others are gazing. Perceiving the gaze of others relies on the integration of multiple social cues, which include perceptual information related to the eyes, gaze direction, head position, and body orientation of others. Autism spectrum conditions (ASC) are characterised by social and emotional deficits, including atypical gaze behaviour. The social-emotional deficits may emerge from a reliance on perceptual information involving details and features, at the expense of more holistic processing, which includes the integration of features. While people with ASC are often able to physically compute gaze direction and show intact reflexive orienting to others’ gaze, they show deficits in reading mental states from the eyes.

**Methods:**

The present study recruited 23 adult males with a diagnosis of ASC and 23 adult males without ASC as a control group. They were tested using a spatial cuing paradigm involving head and body cues in a photograph of a person followed by a laterally presented target. The task manipulated the orientation of head with respect to body orientation to test subsequent shifts of attention in observers.

**Results:**

The results replicated previous findings showing facilitated shifts of attention by the healthy control participants toward laterally presented targets cued by a congruently rotated head combined with a front view of a body. In contrast, the ASC group showed facilitated orienting to targets when both the head and body were rotated towards the target.

**Conclusions:**

The findings reveal atypical integration of social cues in ASC for orienting of attention. This is suggested to reflect abnormalities in cognitive and neural mechanisms specialized for processing of social cues for attention orienting in ASC.

## Background

People generally look in the direction of items that interest them, so following the gaze direction of others helps to reveal their current focus of attention. Therefore, perception of gaze is important for inferring other people’s mental states, such as their interests, goals, or desires [1]. The ability to correctly perceive gaze direction not only plays a role in social interactions, but also in the development of language, theory of mind (ToM), and empathy [2–6].

Gaze direction is a powerful social signal, and it rapidly and reflexively orients observers’ spatial attention in the direction of another’s gaze. This effect has been demonstrated using Posner-like attentional cuing experiments, where a face is first displayed in the centre of the screen and is immediately followed by a target on one side of the screen or the other. A number of cuing experiments have shown faster reaction times to targets when the gaze of the face is directed laterally towards the target, compared to when the facial gaze is directed straight ahead or to the opposite side of the target [7–10]. The fact that this occurs very fast (<200 ms) and when gaze direction is not even predictive of target location suggests the gaze cuing effect is reflexive, automatic and impossible to suppress [8, 10, 11].

Although the eyes provide key information about where people are looking, determining the attention of others often requires the integration of many different social cues. This includes information about gaze direction, head orientation, body posture, and pointing gestures [5, 12, 13]. Each of these signals about social orienting may have mutual effects on our perception of gaze direction. This was illustrated by Langton and Bruce [14], who used a Stroop-like interference paradigm, with head and gaze cues that were in conflict with each other in some conditions. The findings showed symmetrical interference effects by both head and gaze cues on each other, suggesting these components are mutually influential in determining the direction of others’ attention. A study by Bayliss and colleagues [15] used facial displays with eyes where the faces were sometimes rotated by 90 degrees clockwise or counter-clockwise (that is, sideways), such that in these displays the eyes were actually gazing up or down. They found that the rotated faces still produced shifts of observer’s attention to targets appearing on the left or the right of the display. Importantly, the attention shifts occurred towards the direction that the eyes *would* have signalled if the head orientation had been in the normal upright position. This finding shows that when people orient attention based on social cues, gaze direction is referenced to head-orientation.

Information from the eyes alone is not always enough to work out where people are directing their attention. Instead, the relations between different visual cues help to determine the attention of others. For example, when the head is presented in a half-profile with a compatible gaze direction, a typical cuing effect is not observed [9]. Instead, the same gaze direction combined with the head direction facing straight ahead results in speeded responses to targets congruently cued by the gaze direction. The explanation for this effect is that perceiving a head and gaze oriented laterally to the side gives the impression of a person merely facing that direction, without any special intention to direct his or her attention in that direction. However, a forward-directed face combined with averted gaze clearly signifies that something of interest to the side has caught that person’s attention. To make this type of inference about the attention of others requires integrating information from different social cues signalling the gaze direction of others.

**Figure 1:**
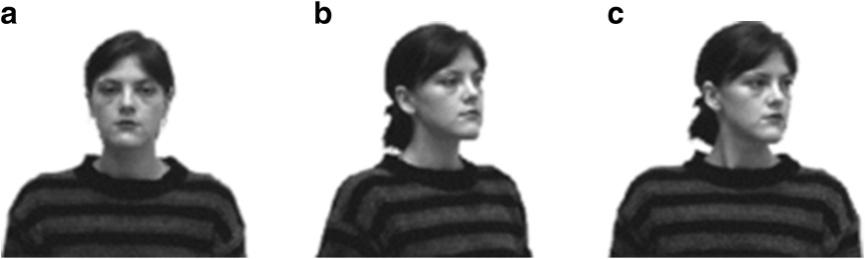
**Examples of the stimuli used in the task.** The examples include: **(a)** a front view of the body and the head, **(b)** both the body and the head oriented 40 degrees laterally, and **(c)** front view of the body with the head oriented 40 degrees laterally.

A similar effect was illustrated by Hietanen [16] in a study investigating how people integrate information from head/gaze cues together with body orientation to facilitate shifts of attention to the gaze direction of others. He showed participants cue stimuli containing a person with different head and body orientations. The social cue was followed by a target that was either congruent or incongruent with the gaze direction signalled by the person in the cue. Participants were faster to detect a target preceded by a person whose head/gaze was averted laterally towards the target, alongside a body position facing straight ahead towards the observer (see Figure 1c) compared to a cue containing a person with both head and body orientation facing straight ahead, towards the participant (see Figure 1a). Importantly, observers also did not show a social orienting effect when targets were preceded by a person whose head and body were *both* averted towards the target (see Figure 1b).

Therefore, perceiving someone with a body facing you along with a head position averted to the side suggests there is something of interest to the side that has grabbed their attention. These findings by Hietanen [16] illustrate that component parts for determining the focus of others’ attention are processed with reference to each other, instead of each having independent effects. A lack of integration between social cues would have been expected to produce even faster social orienting effects for the condition with a cue containing both head and body orientation facing laterally, as both cues were directed towards the target. However, this was not the case, showing that people typically integrate visual component cues for gaze direction to infer the social interest of others. This model for the integration of bodily cues in orienting of attention in directions signalled by others has also been supported by evidence from studies using experimental paradigms other than the Posner-type attention orienting paradigm (that is, a Simon task) [17].

The findings are consistent with various lines of neuroscience research suggesting that social cues about the attention of others are subserved by different neural mechanisms. For example, Perrett and colleagues [13, 18, 19] used single-cell recordings in monkeys and showed different neurons in the STS region selectively code for gaze direction, head orientation and body posture, while still other neurons responded to combinations of eye, head, and body orientation. Consistent with these findings, when the STS region is damaged in monkeys, it produces deficits in making gaze direction judgments, while still leaving other judgments about faces intact [20]. Humans who have acquired damage to the STS region show similar difficulties in making judgments about eye direction [20–23]. Neuroimaging studies have reported separate neural populations coding for pointing fingers [24], for perceiving direct versus averted gaze [25–27], and for coding left versus right oriented gaze directions [28, 29]. There is also a network of brain areas specialised for the visual perception of the body, and this network is different from that involved in perceiving facial information [30]. However, questions remain about how these neural regions that code the various visual components relevant for determining the attention of others interact with each other to facilitate social orienting. Clues to these questions may be obtained from investigating neurodevelopmental conditions involving social disability, such as autism.

High-functioning autism (HFA) and Asperger Syndrome (AS) are autism spectrum conditions (ASC) characterised by difficulties in social and communication functioning alongside repetitive behaviour and restricted interests [31]. Among the main features of these conditions are deficits in understanding others, particularly inferring mental and emotional states [2, 32]. Atypical gaze is common in people with ASC [31, 33, 34], and lack of gaze following is one of the earliest observable behaviours in ASC [35, 36]. People with ASC show deficits in gaze following [37–39], joint attention [4, 40, 41], mutual gaze [42, 43], and they make less eye contact in social situations [44]. While typical controls show expertise for detecting the gaze direction of others using a heuristic involving a dark pupil/iris within a white sclera [45], people with ASC show reduced expertise for the perception of gaze direction, particularly in conditions when eye information is more ambiguous [46].

The difficulties with gaze are thought to be central in the ontogeny of the ToM deficits that characterise those with ASC, as they fail to use gaze direction to infer another’s goals, desires, or points of interest [2, 43, 47]. People with ASC show deficits in the ability to read mental and emotional states from the eye regions [47, 48]. But while people with ASC have difficulties in reading mental states from the eyes, more perceptual aspects of gaze direction appear to be intact. This includes being able to work out geometrically the direction of others’ gaze based on such aspects as their line of site [1, 37, 41]. Many children with ASC show the ability to follow the gaze of others, although this is more evident in higher-functioning individuals [38]. Studies using attentional cuing paradigms have shown intact social orienting to the eye gaze of others in both children and adults with ASC [49–52]. Together, these findings suggest that basic perceptual mechanisms for gaze perception are intact in ASC, but they are unable to use information from the eyes for mental state attributions about others.

A possible explanation for intact performance on certain gaze processing tasks is that those with ASC are utilising perceptual mechanisms that focus more on the featural processing, rather than holistic processing. This idea is consistent with a number of cognitive models proposing that deficits in ASC emerge from difficulties in more holistic types of processing, alongside a greater focus on individual features and small details [32, 53–55]. In the previously described gaze cuing tasks, intact performance could have emerged because people with ASC were only focusing on the eye regions within the displays to determine gaze direction, and not processing the whole stimuli, or integrating information from the facial context and display. Merely being able to follow someone else’s gaze does not necessarily mean taking into account their attention or sharing their experience [38]. Attributing mental states to others is important for gaze-cued attention shifts in adults [56, 57], consistent with evidence from infant studies showing they follow gaze to understand what someone else is attending to (that is, to understand their attentional mental state). Therefore, although people with ASC might follow the gaze direction of others, they may do so in a different manner and utilising different cognitive and neural mechanisms compared to controls. Neuroimaging studies investigating gaze direction processing in ASC have reported that STS activity does not show the same modulation across conditions as seen in controls, suggesting STS activity is not modulated in response to different social meanings attributed to gaze directions in ASC [58–60]. Since the STS contains neural populations specialised for processing different social cues relevant to understanding the gaze direction of others, a lack of modulated STS activity may reflect atypical recruitment of these specialised regions when determining the gaze direction of others in ASC.

The present study investigated the integration of social cues that produce spatial orienting of attention towards lateral targets in ASC. We utilised the same social cue stimuli from a previous study investigating the integration of information derived from both head and body cues for inferring about the attention of others [16]. We investigated cuing effects for both groups with three key conditions of interest: (1) a condition with the social cue containing a person with both her body and head/gaze directed laterally congruent with the target, (2) a condition with the person having her body facing straight ahead alongside the head/gaze directed laterally towards the target, and (3) a neutral reference condition where the social cue contained a person with both her body and head facing straight ahead towards the participant. The responses latencies to conditions 1 and 2 will be compared to the neutral condition to investigate attention orienting by different combinations of head-body cues and, more importantly, to investigate whether attention orienting is cued differently by these head-body cues in individuals with and without ASC.

We expected the control group to show facilitated orienting of attention to combinations of head and body cues inferring someone looking towards something of interest to the side. More specifically, we hypothesised controls would show faster RT’s when the body of the person in the display was facing straight ahead alongside head/gaze direction oriented laterally towards the target in comparison to the neutral reference condition and the condition containing a person whose head and body were *both* averted towards the target. If people with ASC are able to accurately integrate information about attention direction from multiple social cues, then we expect to see results comparable to controls. Alternatively, if those with ASC are unable to accurately integrate information from different sources about the focus of others’ attention, then we expect to see either a lack of social orienting or an abnormal pattern of results compared to controls.

## Methods

### Participants

We recruited 23 adult male participants with ASC (2HFA/21AS: mean age ± standard deviation, 33.9 ± 10.8; Full-Scale IQ, 123.5 ± 13.9) to take part in the research. All participants with ASC had a diagnosis of HFA or AS according to internationally accepted criteria [31, 61] and had been diagnosed in recognized specialist clinics by a psychiatrist, clinical psychologist, or related medical professional (for example, paediatrician, neurologist). Participants had registered to take part in research through the website of our research centre ( http://www.autismresearchcentre.com) and completed the Autism Spectrum Quotient (AQ) as a measure of autistic traits [62]. The AQ is a 50-item forced choice self-report questionnaire asking about behaviours associated with autism. Each question has four response choices and the participant must choose one. The choices include ‘Definitely agree’ , ‘Slightly agree’ , ‘Slightly disagree’ and ‘Definitely disagree’. The questions are worded so that approximately half of them elicit an ‘agree’ response from controls, and half elicit a ‘disagree’ response. Each question is scored one point if it is answered either slightly or definitely towards how a high-functioning person with AS would answer. An example question is ‘I tend to have very strong interests which I get upset about if I can’t pursue’ , which would score 1 if a participant chose either slightly agree or definitely agree. The range of scores is from 0 to 50, with higher scores indicating a greater degree of traits typical of ASC. Previous research has reported mean AQ scores of 35.8 for people with ASC and 16.4 for controls [62].

We also recruited 23 typical adult males as control participants (mean age ± standard deviation, 31.5 ± 11.5; Full-Scale IQ, 121.4 ± 14.3), who had no history of any psychiatric condition and who were recruited from the community. The groups were matched on handedness. All participants completed a measure of intelligence [63] and had normal or adjusted to normal vision. Everyone who took part gave written informed consent, and the study was approved by the Cambridge Psychology Research Ethics Committee. The research was carried out in compliance with the Helsinki Declaration.

### Stimuli

The stimuli were images of a female model displaying a neutral expression and with her head and part of her upper body visible, taken from Hietanen [16]). She was photographed with her gaze direction congruent with her head orientation, so that gaze and head direction were always correlated with each other. Five different pictures were used: a front view of the head and body oriented straight to the camera (see Figure 1a); a front view of the body but with the head oriented 40 degrees to the left and right (see Figure 1c); and the head and body both oriented 40 degrees from the front view to the left and right (see Figure 1b).

The grayscale stimuli were presented on a 20-inch computer monitor with a resolution of 1024 × 768 pixels. The height of the stimuli was 9.5 cm on the screen. The target was an asterisk measuring 0.5 cm on the screen. As the participants were seated approximately 57 cm from the computer screen, the stimulus and asterisk measured 9.5 and 0.5 degrees of visual angle, respectively. The stimulus presentation and data collection were controlled by DMDX [64] running on an Inspiron laptop PC and connected to the monitor. A response box was used to collect responses, and was specially constructed to be compatible with the computer and DMDX.

### Procedure

Participants were seated comfortably in a quiet and dimly lit testing room, with the computer screen situated at eye level. Each trial of the experiment began with a fixation-cross shown for 750 ms, followed immediately by one of the head-body stimuli displayed on the screen for 50 ms.

The fixation cross was located in the middle of the pictures, which was approximately in the middle of the chin in the front view picture, and at the same height but in the chin/neck area in the profile pictures. After the presentation of the stimulus picture, the target appeared 5.6 cm to either the left or right side of the fixation point until the response was executed. The stimulus onset asynchrony (SOA), which was the time between the onsets of the head-body stimulus and the reaction signal, was jittered between 150 and 250 ms to avoid repetitive presentation and responding. Since these times were not of interest to the present study and because no differences were found in the cuing effect among the SOA’s in previous research [16], we did not include time as a factor in the analyses. Following the participant’s response, a blank screen appeared for 1000 ms, after which the next trial began.

Participants placed the index finger of their dominant hand on the button of a response box and were instructed to press the button as soon as possible after detecting the target. They were told the orientation of the head and the body in the pictures did not predict the side where the target would appear. By combining the cue stimuli with the two target locations (left and right), the directional congruency between the different types of cues and reaction signals was varied. This produced five different stimulus conditions:

1. the body straight/head straight condition included a front view of the head and body followed by a target appearing either to the left or the right of the cue, that is,, the neutral condition;
2. the body congruent/head congruent condition involved the head and body both oriented 40 degrees to the left or the right followed by a target appearing on the same side the head and body were oriented towards;
3. the body incongruent/head incongruent condition involved the head and body both oriented 40 degrees to the left or right followed by a target on the opposite side the head and body were oriented towards;
4. the body straight/head congruent condition included a front view of the body along with the head oriented 40 degrees to the left or right, followed by a target appearing on the same side the head was oriented towards;
5. the body straight/head incongruent condition included a front view of the body with the head oriented 40 degrees to the left or right followed by a target appearing on the opposite side the head was oriented towards.

Different stimulus conditions were presented equiprobably in a random order. There were 200 test trials in the experiment, with 40 trials per condition. In order to discourage anticipatory responses, 20 ‘catch’ trials were added. On these trials, no reaction signals were presented, and the subjects were instructed not to press the response button. This created a total number of 220 trials, which were presented in two blocks separated by a short resting period. Each block took approximately 4 to 4 1/2 minutes to complete. Prior to the experimental trials, all participants were trained on the task by completing 15 practice trials and 3 catch trials.

## Results

Response times below 100 ms and above 1,000 ms were removed from the data, and accounted for <2% of the data. The means and standard deviations (SD) for all conditions are presented in Table 1. There were no significant differences between the groups for age, *t*(44) = 0.74, *P* = .463, or for full-scale IQ, *t*(44) = 0.50, *P* = .620. The AQ scores for the sample with ASC (N = 23, mean AQ score = 38.9, SD = 5.6, 91.3% scoring 32+) were very similar to those observed in previous studies (N = 58, mean AQ score = 35.8, SD = 6.5, 80% scoring 32+; [62]).

**Table 1:** **Mean scores (SD) for the group characteristics and experimental conditions across both groups**

Group	Group characteristic			Experimental condition				
	Age	FSIQ	AQ	BS/HS	BS/HC	BC/HC	BS/HI	BI/HI
ASC	33.9 (10.8)	123.5 (13.9)	38.9 (5.6)	331.6 (40.3)	330.8 (40.9)	324.3 (44.2)	329.2 (41.5)	329.8 (41.0)
Control	31.5 (11.5)	121.4 (14.3)	na	311.6 (52.6)	301.8 (51.4)	308.9 (53.9)	309.8 (52.6)	310.7 (53.4)

BS/HS, body straight/head straight; BS/HC, body straight/head congruent; BC/HC, body congruent/head congruent; BS/HI, body straight/head incongruent; BI/HI, body incongruent/head incongruent.

A general linear model ANOVA with repeated measures was performed on the mean response latencies to detect a target with Condition (see 5 conditions above) as the within-subject factor and Group (Controls versus ASC) as the between-subject factor. Post hoc t-tests with Bonferroni corrections were done where appropriate. The results revealed a main effect of Condition, *F*(4, 41) = 3.08, *P* = .026. However, with Bonferroni corrections (p value of .05/10 = .005) none of the comparisons reached significance (all *P*’s > .005), although there was a trend towards significance for the body straight/head congruent condition (Mean = 316.2; SD = 48.1) to be faster than the body straight/head straight condition (Mean = 321.6; SD = 47.0), *t*(45) = 2.79, *P* = .008. There was no main effect of Group, *F*(1, 44) = 2.24, *P* = .142.

**Figure 2:**
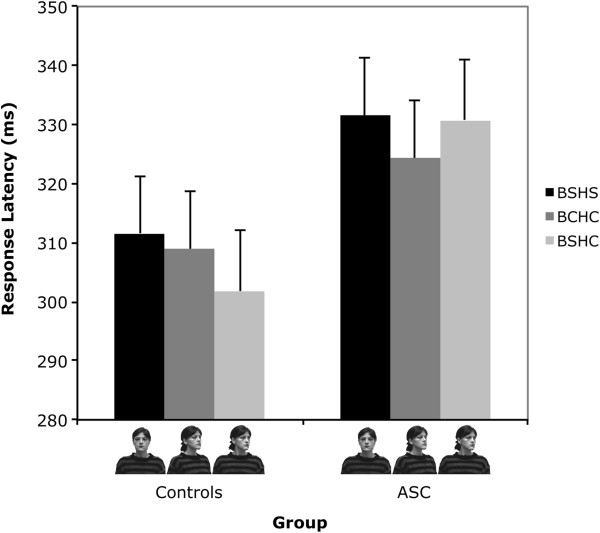
**Mean response latencies (ms) in the three experimental conditions of interest for the autism spectrum condition (ASC) and control groups.** Error bars represent standard error of the mean. In the legend, BS/HS refers to the body straight/head straight condition; BC/HC refers to the body congruent/head congruent condition; and BS/HC refers to the body straight/head congruent condition.

Importantly, there was also an interaction between Condition and Group, *F*(4, 41) = 4.33, *P* = .007. Planned comparisons were carried out using within-subject paired-samples t-tests between the three conditions of interest (body straight/head congruent versus body straight/head straight versus body congruent/head congruent). For the control group, the response latencies for the body straight/head congruent condition were significantly faster than the body straight/head straight, *t*(22) = 2.89, *P* = .008, and the body congruent/head congruent condition, *t*(22) = 2.69, *P* = .013 (see Figure 2). There was no significant difference between the body straight/head straight and the body congruent/head congruent conditions, *t*(22) = 1.05, *P* = .307.

For the ASC group, response latencies for the body congruent/head congruent were significantly faster than both the body straight/head straight condition, *t*(22) = 2.91, *P* = .008, and the body straight/head congruent condition, *t*(22) = 2.44, *P* = .023 (see Figure 2). There was no significant difference between the body straight/head straight and the body straight/head congruent conditions, *t*(22) = 0.71, *P* = .481.

Unplanned comparisons were also carried out between all other conditions within each group with Bonferroni corrections (*P* value of .05/7 = .007). Results showed that for the control group the body straight/head congruent condition was faster than both the body straight/head incongruent condition, *t*(22) = 3.21, *P* = .004, and the body incongruent/head incongruent condition, *t*(22) = 4.11, *P* = .000. There were no other significant differences between any of the conditions for either the control or ASC group (all *P*’s > .007). Error rates and missed trials for the experiment were very low (<5% overall), and did not differ between the groups for either the experimental or catch trials (*P* > .05 for both).

## Discussion

Consistent with previous research [16, 17], the controls showed reflexive shifts of attention to the combination of a body position oriented straight ahead towards the observer alongside head/gaze direction averted laterally towards a target, compared to when both the body and head orientation were both directed straight ahead. The time-course of the task with a very short SOA suggests the mechanisms involved reflexive orienting of attention [8]. The attentional orienting effect produced by social cues having an averted head/gaze along with a body facing straight ahead suggests the control group interpreted the mental state of that person. More specifically, they may have inferred that person oriented their attention towards something of interest to the side, which shifted their attention in the same direction as that persons gaze. The condition with the person in the cue having both their head/gaze direction and body orientation averted towards the target did not facilitate attention orienting for the control group. The controls may have inferred that the person in that condition was merely rotated in that orientation, and not likely attending to something of interest. This is consistent with previous experimental studies showing that attributing mental states to others facilitates gaze-cued attention orienting [56, 57]. In those studies, different mental state attributions made by the observers determined whether or not they oriented their attention to follow the gaze of others. From results such as these it has been proposed that gaze-following involves two separate but parallel mechanisms; one mechanism that relies on perceptual stimulus-driven properties about determining gaze direction, and another that utilises the attribution of underlying mental states [9, 56]. These mechanisms are similar to the mechanistic and mentalistic modes of perceiving the gaze direction of others [7, 65–67]. Results from the present study suggest the control group mainly utilised the mentalising mechanism during gaze-following.

The ASC group did shift their attention in the direction of another person, but it was based on an atypical combination of head/gaze and body cues of another person compared to the control group. They did not have speeded responses when the combination of visual signals suggested another person was attending laterally towards something of interest, compared to the neutral condition. Instead, the ASC group showed facilitated shifts of attention when the social direction cues from the head and body were both oriented laterally towards the target location. The group differences were not attributable to general deficits in attention orienting by social cues in ASC, as there was no group difference overall in the task.

The results of atypical integration of social cues in the ASC group suggest they may have been relying more on the mechanistic mode when perceiving cues about the gaze direction of others, rather than relying on mentalistic mechanisms. This interpretation of the results in terms of reduced use of mentalising mechanisms is consistent with studies showing reduced use of gaze direction information for inferring the intentions of others in ASC. For example, in the ‘smarties task’ [68], a picture of someone surrounded by four types of candy is shown and participants are asked which candy the person wants. While control children say the person in the picture wants the candy they are looking towards, children with ASC respond with random choices or choose the candy they prefer. A study investigating the prediction of actions from perceiving the gaze direction of actors revealed that people with ASC had similar performance in action anticipation across both social and non-social conditions, while the anticipations of controls were influenced only in the social condition [65]. This result was interpreted as showing those with ASC were utilising an atypical strategy for attributing intentions to gaze direction, based on lower-level and non-social features. This result would be consistent with the ASC group utilising the mechanistic mode of perceiving the gaze direction of others [7, 65–67].

The atypical integration of social cues to shift attention to the gaze direction of another person by the ASC group in the present study may have emerged from using a greater reliance on mechanistic properties about gaze direction. This could have involved either atypical processing of social cues or from perceiving non-social elements within the social stimuli. Those with ASC could have been focusing on only one individual feature of the social cues during the task. The condition where the ASC group showed a facilitated response compared to the neutral condition was the only condition where body orientation was directed towards the target. So if they focused only on body orientation during the task, this could potentially have produced the results. This explanation would be consistent with psychological theories of ASC about enhanced perception of features and details at the expense of more holistic processing [32, 53–55]. For example, people with ASC show strengths in tasks where they are required to perceive shapes in terms of their features, such as the embedded features task [69, 70] and the block design [71]. An eye-tracking study using scenes of social interactions reported that children with ASC spontaneously focused more attention on the bodies of people in the scenes than controls, and less focus was paid to facial features [72]. Therefore, the atypical integration of social cues by the ASC group in the present study may have emerged from using a different viewing strategy to controls, where they focused solely on one individual feature of the social cue to facilitate shifts of attention.

Alternatively, the ASC group may have been responding to non-social aspects of the stimuli when perceiving the direction of gaze. Some have suggested those with ASC use ‘directional properties’ or transient motion signals, rather than gaze cues [73]. If they were focusing mainly on the body orientation during the experiment, they may have utilised directional cues signified by the direction the body was facing. This could have involved a number of possible low-level visual differences between the stimuli across conditions, such as shading, line orientation, or small visual details. Previous social orienting studies have shown that non-social stimuli might have similar abilities to facilitate shifts of attention as eyes in people with ASC [51, 74]. This explanation of the results would still represent atypical integration of social cues for shifting attention, although it would involve those with ASC focusing on non-social visual features of the stimuli.

The present findings are consistent with neuroimaging research in ASC reporting atypical STS activity across different gaze direction processing conditions [58–60]. There are neurons within the STS region that code for different visual cues, such as gaze directions, head orientations, and body positions [13, 19, 75]. Studies have reported that STS activity in control groups is normally modulated based on social meanings attributed to eye gaze direction across conditions, with greater STS activity when conditions require greater attribution of intentions or social significance. This modulation in activity of the STS region across conditions involving different gaze direction attributions is reported to be either absent in ASC [59, 60], or the pattern of STS activity is reversed such that greater activity is seen to non-social cues and reduced to gaze cues [58]. These differences in brain activity occur despite similar behavioural performance between the groups during the tasks. The neuroimaging results show that the neural mechanisms for processing gaze direction are being utilised differently in ASC, with reduced STS activation when social meaning is attributed to gaze direction [58, 59]. Therefore, the STS may be less sensitive to the social meaning of eye gaze in ASC [59, 60], and the functional specialisation of subregions within the STS may be disrupted or less effective [61].

A limitation of the present study is that we did not include children, females, or lower functioning participants with ASC, so the findings may not generalise across the autism spectrum. However, we find it interesting that even though our participants were all high-functioning adults, they still displayed atypical social orienting to the gaze of others in a simple lab-based experimental task. Some have suggested that people with ASC who are older and more intelligent may show normal performance on lab tests of social functioning, including judgments of gaze direction [73]. This was not the case in the present study, suggesting that atypical social processing of the gaze direction of others may be a central feature of ASC. However, research in children at low and high risk of developing ASC has reported that poor gaze following may not specifically predict later diagnosis of ASC [76]. In addition, differences in non-social attention processes may be early features of ASC [77, 78]. The present study did not include a non-social condition in the task in order to test whether the results are specific to the processing of social information or whether they may reflect more general attentional differences. Further research with this paradigm is warranted in a wider group of participants across the autism spectrum and should include non-social conditions. No differences were found for response latencies to the incongruent conditions compared to the neutral condition, which may not have been expected. However, this pattern of results is considered typical for reflexive attention orienting by uninformative peripheral cues [79]. This pattern has also been reported previously in several studies using gaze direction cues [8, 80], and also in a previous study using the same stimuli as the present study [16]. The present experiment did not include a condition with the social cue having a body facing laterally towards the target and the head oriented straight ahead towards the participant, which would have directly tested whether people with ASC were focusing solely on the body orientation to shift their attention to the head/gaze direction of others.

## Conclusions

The results of the present study show atypical orienting to the attention of others in ASC. Findings suggest a reliance on the perception of visual components about direction of others’ attention, and impaired integration of social cue information to infer that people have turned their attention towards a target, rather than simply looking in some direction. These results are consistent with neuroimaging studies showing atypical modulation of the STS across conditions.

## Abbreviations

AQ: Autism-Spectrum Quotient
AS: Asperger syndrome
ASC: autism spectrum conditions
HFA: high-functioning autism
SOA: stimulus onset asynchrony
ToM: Theory of mind.
